# Short chain fatty acid, acetate restores ovarian function in experimentally induced PCOS rat model

**DOI:** 10.1371/journal.pone.0272124

**Published:** 2022-07-26

**Authors:** Kehinde S. Olaniyi, Al-amin M. Bashir, Stephanie E. Areloegbe, Isaiah W. Sabinari, Christopher O. Akintayo, Adesola A. Oniyide, Ayodeji Aturamu

**Affiliations:** 1 Department of Physiology, Cardio/Repro-metabolic and Microbiome Research Unit, College of Medicine and Health Sciences, Afe Babalola University, Ado-Ekiti, Nigeria; 2 HOPE Cardiometabolic Research Team & Department of Physiology, College of Health Sciences, University of Ilorin, Ilorin, Nigeria; West China Second University Hospital, Sichuan University, CHINA

## Abstract

**Background:**

Polycystic ovarian syndrome (PCOS) is pathogenically characterized with hyperandrogenism and metabolic alterations, which often result in ovarian changes and infertility in women of reproductive age. Epigenetic changes have been linked to the development of PCOS. However, the involvement of epigenetic regulator, histone deacetylase (HDAC) in PCOS-driven ovarian dysfunction is not clear. Howbeit, the present study hypothesized that acetate, an HDAC inhibitor (HDACi) would protect against ovarian dysfunction in experimentally induced PCOS.

**Materials and methods:**

Female Wistar rats weighing 120–150 g were randomly divided into four groups (n = 6). The groups received vehicle, sodium acetate (200 mg/kg), letrozole (1 mg/kg) and letrozole with acetate by oral gavage respectively. The administrations were done daily for 21 days.

**Results:**

The rat model of PCOS had increased body weight and ovarian weight, 1-hr postload glucose and plasma insulin, testosterone and LH/FSH ratio as well as reduced insulin sensitivity and plasma 17-β estradiol and sex hormone binding globulin. This model of PCOS in addition showed a significant increase in plasma and ovarian triglyceride, total cholesterol, TNF-α and HDAC, and ovarian malondialdehyde as well as a significant reduction in ovarian glutathione peroxidase/reduced glutathione and NrF2 with the histology of ovarian tissues showing disrupted morphology with significant increase in the number of degenerated follicles compared with control group. These alterations were however attenuated when treated with HDACi, acetate.

**Conclusion:**

Altogether, the present results suggest that acetate protects ovarian function with evidence of normal growing follicles and enhanced circulating 17-β estradiol by inhibition of HDAC.

## 1. Introduction

Polycystic ovarian syndrome (PCOS) is a complex endocrine disorder of public health concern that devastates between 6–21% of reproductive aged females worldwide [1]. Clinically, a male-phenotype hyperandrogenism, dysmenorrhea, insulin resistance (IR) and multicystic ovaries are markedly recognizable features that sum up to infertility and a heightened risk of ovarian and/or endometrial cancers in PCOS women [2,3]. The etiology and mechanisms pertaining to the reproductive and metabolic disturbances associated with PCOS have however not been clearly understood making treatment complex [4]. Hence, there is an overarching need to probe into pathophysiological events that underlie the central endocrine dysfunctions and local metabolic imprints of the ovaries in PCOS.

The network of metabolic derangements observed in patients and experimental models of PCOS include IR, hyperglycemia, hyperlipidemia and chronic inflammation [5]. It has been reported that IR with or without obesity precedes these changes, being expressed in 60–95% of total PCOS cases [2]. Moreover, with excessive weight gain/obesity and a higher body mass index, altered insulin sensitivity can interact with hyperandrogenism to aggravate PCOS phenotypes [6]. Insulin resistance consequentially raises insulin level and prevents insulin suppression of fat lipolysis leading to peripheral lipid deposition, lipotoxicity and activated pro-oxidant/inflammatory pathways with non-adipose organs as key victims [7]. Existing evidence have shown ovarian TG accumulation and uric acid-mediated inflammation in connection with overweight in PCOS animal models [8,9]. Furthermore, IR-hyperinsulinemia could contribute to anovulation through stimulatory effects of luteinizing hormone (LH) on androgen production in the ovarian theca cells and via inhibition of sex hormone-binding globulin (SHBG) production by hepatocytes [10].

Aberrant inflammatory condition such as elevated tumor necrosis factor-α (TNF-α) in part mediates the complex unfavorable reproductive-metabolic nexus of IR, lipotoxicity and ovarian dysfunction in PCOS. Tumor necrosis factor-α (TNF- α) triggers some protein kinases that activate serine/threonine phosphorylation instead of the usual tyrosine phosphorylation of insulin receptor substrate, thus inhibiting the insulin transduction pathway [11]. Similarly, elevated ovarian TNF-α contributes to tumor angiogenesis and ovarian cancers [11,12]. There is a complex interplay between several environmental, genetic and epigenetic factors driving the PCOS dysmetabolic/pro-inflammatory environment. Epigenetic modifications regulate transcriptional response of inflammatory genes through addition and deletion of functional groups from chromatin structures in response to various endocrine and metabolic precursors [13]. The removal of acetyl group from lysine residues of histone and non-histone proteins by histone deacetylase (HDAC) results in alteration of gene expression through deconstruction of chromatin structure, with subsequent access by transcription factors [14]. Epigenetic checkpoints are targets for therapeutic interventions, therefore, knockdown of HDAC could be essential in alleviating TNF-α-worsened oxidative/inflammatory stress and ovarian dysfunction, although there are insufficient studies providing precise information on this pathway with regards to PCOS.

The Nuclear Factor (Erythroid-Derived 2)–Like 2 (Nrf2) is a transcription factor and master regulator of antioxidant genes through interaction with nuclear antioxidant response element (ARE) leading to the expression of key antioxidants necessary for cytoprotection against reactive oxygen species (ROS) [15]. Polycystic ovary syndrome-related oxidative imbalance involves a down regulated Nrf2 which permits oxidative stress, and inflammatory response [16]. Glutathione (GSH) is an important thiol-based tripeptide involved in detoxification of free radicals and superoxides [17]. The glutathione-dependent antioxidant system consisting of glutathione peroxidase (GSH-Px), glutathione S-transferase (GSH-ST) and glutathione reductase (GSH-Rd) forms a crucial wing of cellular antioxidant mechanisms that prevents the injurious effects of lipotoxicity/ROS associated with IR-related diseases [18]. Glutathione peroxidase, a cytoplasmic and mitochondrial-resident enzymatic antioxidant scavenges superoxides such as hydrogen peroxide (H_2_O_2)_ to water (H_2_O), causing GSH to become oxidized (GSSG). Nicotinamide adenine dinucleotide phosphate provides the reducing power that replenishes cells with GSH via the action of GSH-Rd on GSSG to sustain protection against excessive ROS attack which is an important requirement for oocyte maturation and survival [18].

Aside lifestyle adjustment, oral contraceptives and metformin are the first line pharmacotherapy for women with PCOS [19]. Treatment with oral contraceptives have previously managed hormonal imbalance, while treatment with metformin has effectively reduced circulating glucose, insulin and androgen levels thereby improving whole body metabolic status although there are some controversies around these interventions [4,19]. Growing research evidence has revealed that upper gut microbiota-derived short chain fatty acids (SCFAs), acetate, propionate and butyrate are modulators of immune and metabolic functions. Through G protein-coupled receptor binding, SCFAs improve systemic and intra-mural inflammatory and metabolic profile. Of the three most abundant SCFAs, studies have extensively reported butyrate and propionate as the inhibitors of HDAC [15,20,21]. Nevertheless, acetate has been found to repress HDAC activity in T cells [22,23], and in our recent studies in diabetic animal models [24,25]. However, the effect of acetate on HDAC-dependent ovarian dysfunction in PCOS pathobiology is not known. In this study, administration of letrozole, an aromatase inhibitor experimentally induced an androgenized systemic and ovarian milieu that mimics clinical PCOS manifestations. Howbeit, the present study hypothesized that acetate, an HDAC inhibitor (HDACi) would protect against ovarian dysfunction in experimentally induced PCOS.

## 2. Materials and methods

### 2.1 Experimental design

Female Wistar rats weighing 120–150 g were procured from the animal house of the College of Health Sciences, Afe Babalola University, Ado-Ekiti. These rats had unlimited access to standard rat chow and tap water and the study was conducted in accordance with the National Institutes of Health Guide for the Care and Use of Laboratory Animals, and approval to carry out the study was obtained from the Institutional Ethical Review Board of the university with the approval number (ABUADERC/15/2021), and effort was made to minimize the number/suffering of the animals used. All the animals chosen for the work were with at least three consecutive regular estrous cycles and on the same estrous phase, which was determined via vaginal smear for each rat. After acclimatizing the animals for two weeks, they were randomly divided into four groups (n = 6/group) namely: Control, acetate-treated (NaAc), letrozole-treated (PCOS) and PCOS+NaAc groups. Rats were maintained under standard environmental conditions of temperature (22–26°C), relative humidity (50–60%), and 12-hour dark/light cycle. Polycystic ovarian syndrome was experimentally induced by administering letrozole (1 mg/kg) once daily for 21 days as previously reported [8,26–28].

### 2.2 Treatment

Distilled water was given as vehicle to the control group, NaAc group received 200 mg/kg of sodium acetate (Sigma-Aldrich, St Louis, MI) [24,29], PCOS group received 1 mg/kg of letrozole (Sigma-Aldrich, St Louis, MI) and PCOS+NaAc group received letrozole and sodium acetate. The administrations were done uninterruptedly by oral gavage for 21 days [8,26,27]. Initial and final body weights were monitored, and the body weight gain was estimated.

### 2.3 Glucoregulatory indices

The 1-hour postload glucose was determined 48 hours before the sacrifice of the rats. After 12-h overnight fast, basal blood glucose was determined, and the rat were loaded with glucose (2 g/kg; oral gavage). Then blood was obtained at 60 minutes. Blood glucose levels were monitored with a hand-held glucometer (ONETOUCH-LifeScan, Inc., Milpitas, CA, USA). Insulin sensitivity was determined using quantitative check for insulin sensitivity (QUICKI) as previously described [9,30].

### 2.4 Collection of samples

At the end of the treatment, the rats were sacrificed by intraperitoneal injection of sodium pentobarbital (50 mg/kg). Blood was collected by cardiac puncture into heparinized tube and centrifuged at 704 *g* for 5 min at room temperature. Plasma was stored frozen until the time of biochemical analysis.

### 2.5 Preparation of ovarian tissue homogenate

After weighing the ovary, 100 mg section of the tissue was carefully removed and homogenized with a glass homogenizer in phosphate buffer solution and centrifuged at 8000 g for 10 min at 4°C and the supernatant was collected and frozen stored until the time of biochemical analysis.

### 2.6 Biochemical analysis

#### 2.6.1 Plasma endocrine profile

Plasma insulin, 17-β estradiol, testosterone, luteinizing hormone (LH), follicle stimulating hormone (FSH) and sex hormone binding globulin (SHBG) concentrations were determined with Rat ELISA kits obtained from Calbiotech Inc. (Cordell Ct., El Cajon, CA 92020, USA) and the manufacturer’s procedures were followed. The ratio of LH/FSH was estimated.

#### 2.6.2 Plasma and ovarian lipid profile

Standard colorimetric methods using assay kits obtained from Fortress Diagnostics Ltd. (Antrim, UK) were used to determine the triglyceride (TG), total cholesterol (TC) and free fatty acid (FFA). from the plasma and ovarian tissue.

#### 2.6.3 Ovarian lipid peroxidation and antioxidant markers

Malondialdehyde (MDA) was determined from the ovarian tissue by standard non-enzymatic spectrophotometric method using assay kits from Randox Laboratory Ltd. (Co. Antrim, UK). This method involves the reaction of MDA in the sample with thiobarbituric acid (TBA) to generate an MDA-TBA adduct, which was quantified spectrophotometrically, while glutathione (GSH) was determined using a non-enzymatic spectrophotometric method with assay kits obtained from Oxford Biomedical Research Inc. (Oxford, USA). Glutathione (GSH) concentration was determined by spectrophotometric method based on the oxidation of GSH in the sample by the sulfhydryl reagent 5,5′-dithio-bis (2-nitrobenzoic acid) (DTNB) to form the yellow derivative 5′-thio-2-nitrobenzoic acid (TNB), measured at 412 nm. Glutathione peroxidase (GPx) was determined from the ovarian tissue by standard enzymatic spectrophotometric method using assay kits from Oxford Biomedical Research Inc. (Oxford, USA) and in adherence to manufacturers’ assay guidelines.

#### 2.6.4 Nuclear factor erythroid-derived 2–like 2

The ovarian level of Nrf2 was determined by the quantitative standard sandwich ELISA technique using monoclonal antibody specific for this parameter with the rat kit obtained from Elabscience Biotechnology Inc. (Wuhan, Hubei, P.R.C., China) and in compliance with the manufacturer’s assay procedures.

**2.6.5 Plasma and ovarian tumor necrosis factor-α.** The levels of TNF-α were determined in the blood and ovarian tissue by quantitative standard sandwich ELISA technique using a monoclonal antibody specific for these parameters with rat kits obtained from Elabscience Biotechnology Inc. (Wuhan, Hubei, P.R.C., China).

#### 2.6.6 Plasma and ovarian histone deacetylase

The plasma and ovarian tissue levels of HDAC were determined using Rat ELISA kits obtained from Bioassay Technology Laboratory (Yangpu Dist. Shanghai 200090, China)., in compliance with the manufacturer’s procedure.

### 2.7 Histological assessment of ovaries

For histomorphological evaluation using hematoxylin and eosin (H & E) staining technique, a section of the ovary was fixed in 10% formolsaline overnight and thereafter dehydrated, embedded in paraffin and sectioned at 5-μm thickness. The slides were prepared and examined using OPTO-Edu industrial camera light microscope and a computer (Nikon, Japan).

### 2.8 Stereological assessment of ovarian follicles

For the stereological evaluation of ovarian follicles, ten sagittal sections of the ovary from each animal were analyzed in a serial section. Images of the ovarian follicles were captured systematically using an OPTO-Edu industrial camera light microscope and a computer. The sections (5-μm widths) were captured and then processed with an image-processing and analysis software Image-J (Version 1.52). The mean total number of degenerated follicles per section was determined after counting every ten sections as previously reported [28,31].

### 2.9 Data analysis and statistics

The distribution of the data was confirmed using Shapiro-Wilk test and the data were normally distributed. All data were expressed as means ± SD. Statistical group analysis was performed with GraphPad Prim software version 5. One-way ANOVA was used to compare the mean values of variables among the groups. Bonferroni’s test was used for *post hoc* analysis and statistically significant differences were accepted at p less than 0.05.

## 3. Results

### 3.1 Impact of sodium acetate on body and ovarian weight in LET-induced PCOS rat model

**Fig 1:**
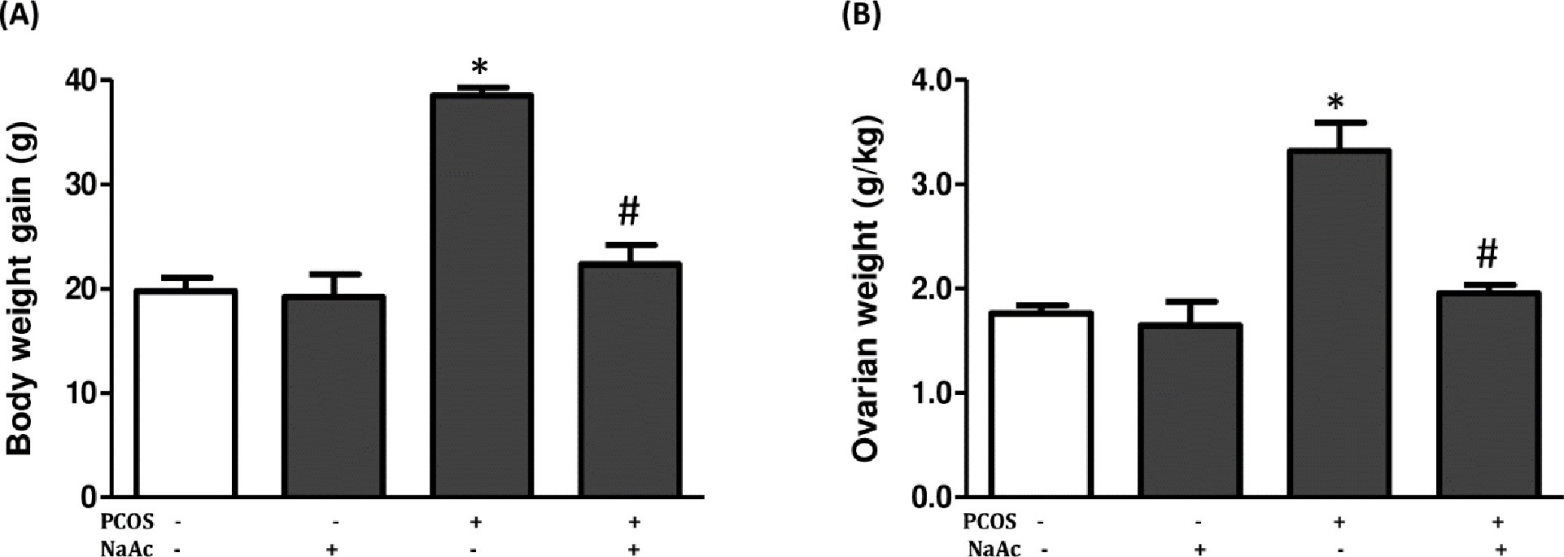
Impact of sodium acetate on body weight (a) and ovarian weight (b) in LET-induced PCOS rat model. Data are expressed as mean ± SD. n = 6. Data were analyzed by one-way ANOVA followed by Bonferroni *post hoc test*. (*p<0.05 VS. CTL; ^#^*p*<0.05 VS. PCOS). Control (CTL); Polycystic ovarian syndrome (PCOS); Sodium acetate (NaAc).

Group of animals with PCOS significantly had increased (p<0.05) body weight and ovarian weight when compared to the control animals and administration of sodium acetate significantly decreased (p<0.05) the body weight and ovarian weight in PCOS+NaAc group compared to the untreated PCOS group (Fig 1).

### 3.2 Impact of sodium acetate on ovarian histomorphology in LET-induced PCOS rat model

**Fig 2:**
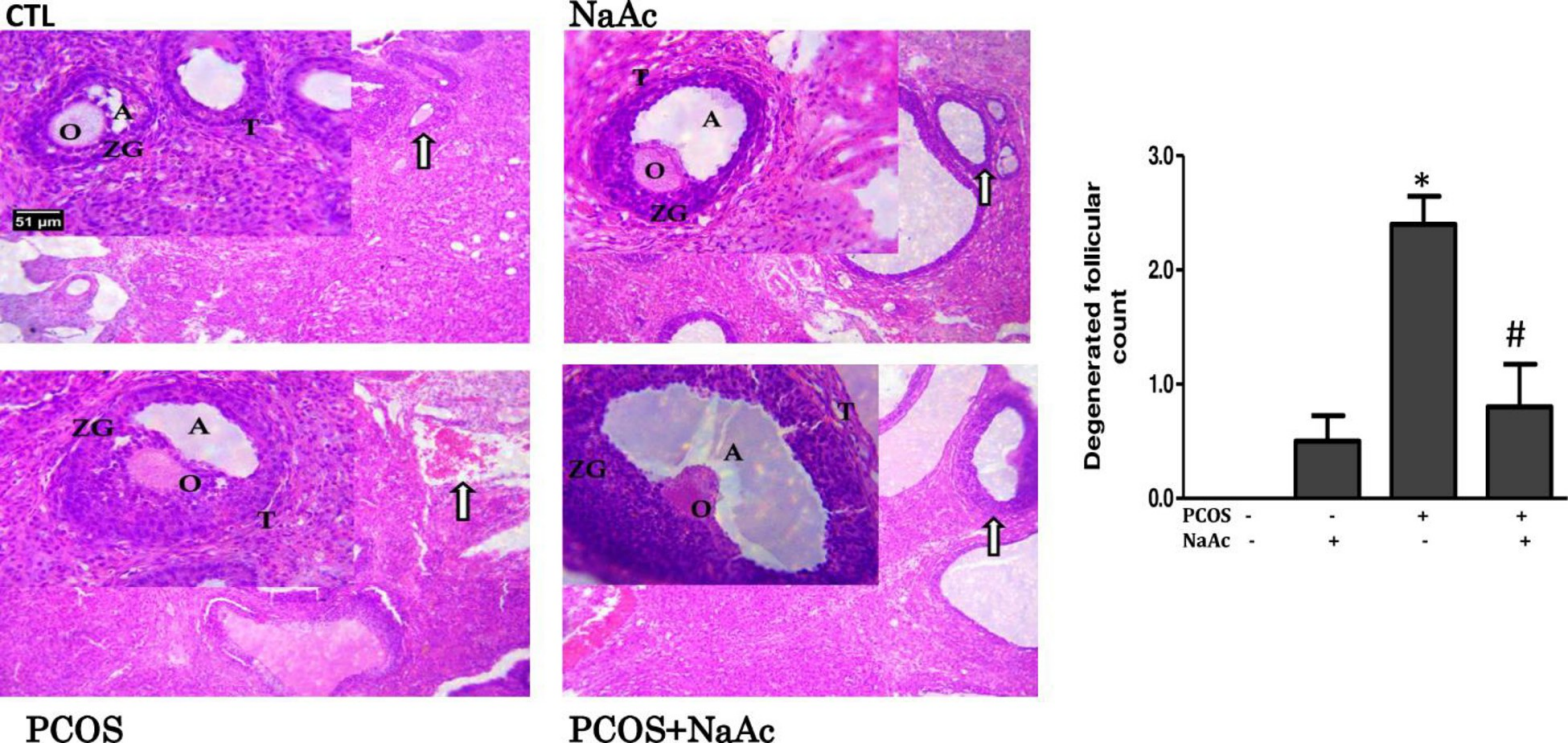
Impact of sodium acetate on the histomorphology of ovaries in LET-induced PCOS rat model. The photomicrographs of ovarian tissues show ovarian tissue with normal follicles and normal antrum, granulosa cells, thecal cells and oocyte in control animals (CTL), ovarian tissue with normal follicles and normal granulosa cells, thecal cells, oocyte and large antrum in NaAc-treated animals (NaAc), ovarian tissue with degenerated follicles and disrupted granulosa cells, thecal cells, antrum and oocyte in PCOS animals (PCOS) and ovarian tissue with preserved follicles and normal granulosa cells, thecal cells, oocyte and large antrum in PCOS+NaAc-treated animals; (H & E paraffin stain; transverse section; Scale bar 51 μm). Oocyte (O); Antrum (A); Thecal cells (T); Granulosa cell (ZG). Data are expressed as mean ± SD. n = 6. Data were analyzed by one-way ANOVA followed by Bonferroni *post hoc test*. (*p<0.05 VS. CTL; ^#^*p*<0.05 VS. PCOS). Control (CTL); Polycystic ovarian syndrome (PCOS); Sodium acetate (NaAc).

Histological analysis of ovarian tissues showed degenerated follicles and disrupted granulosa cells, thecal cells, antrum, and oocyte in PCOS animals compared with normal follicles and normal antrum, granulosa cells, thecal cells and oocyte in control animals. However, the ovarian tissues of PCOS+NaAc animals showed preserved follicles with normal granulosa cells, thecal cells, oocyte and large antrum. In addition, the number of degenerated follicles was significantly higher (p<0.05) in PCOS animals compared to the control animals and this was significantly decreased (p<0.05) in PCOS+NaAc animals compared to the untreated PCOS animals (Fig 2).

### 3.3 Impact of sodium acetate on endocrine profile in LET-induced PCOS rat model

Plasma testosterone and LH/FSH ratio increased while 17-β estradiol and SHBG decreased significantly (p<0.05) in animals with PCOS compared to the control animals, which were significantly reduced and increased (p<0.05) respectively in PCOS+NaAc animals compared to the untreated PCOS animals (Table 1).

**Table 1:** Impact of sodium acetate on endocrine profile in LET-induced PCOS rat model.

GROUPS	CTL	NaAc	PCOS	PCOS+NaAc
Testosterone (ng/mL)	0.26 ± 0.11	0.24 ± 0.10	2.66 ± 0.51*	1.13 ± 0.05*^#^
17-β estradiol (ng/mL)	5.03 ± 0.40	4.77 ± 0.52	1.71 ± 0.26*	4.63 ± 0.84^#^
LH/FSH ratio	18.58 ± 0.83	16.99 ± 0.53	34.03 ± 3.91*	20.66 ± 0.63^#^
SHBG (pg/mL)	530.80 ± 3.23	548.8 ± 10.53	246.5 ± 12.29*	428.30 ± 18.39^#^

Data are expressed as mean ± SD. n = 6. Data were analyzed by one-way ANOVA followed by Bonferroni *post hoc test*.

(*p<0.05 VS. CTL

^#^*p*<0.05 VS. PCOS). Control (CTL); Polycystic ovarian syndrome (PCOS); Sodium acetate (NaAc).

### 3.4 Impact of sodium acetate on glucoregulatory indices in LET-induced PCOS rat model

**Fig 3:**
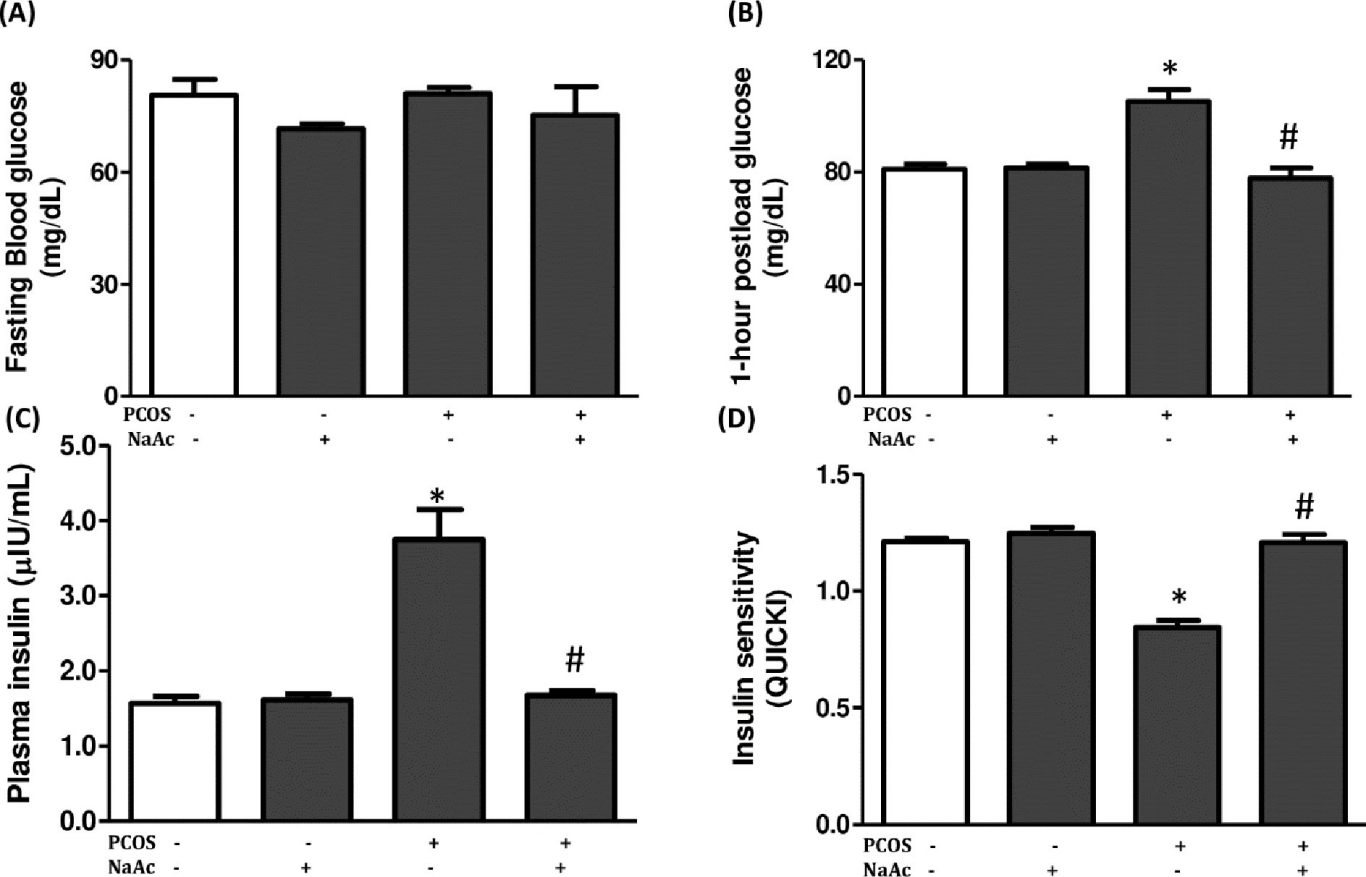
Impact of sodium acetate on fasting blood glucose (a), 1-hour postload glucose (b), fasting insulin (c) and insulin sensitivity (d) in LET-induced PCOS rat model. Data are expressed as mean ± SD. n = 6. Data were analyzed by one-way ANOVA followed by Bonferroni *post hoc test*. (*p<0.05 VS. CTL; ^#^*p*<0.05 VS. PCOS). Control (CTL); Polycystic ovarian syndrome (PCOS); Sodium acetate (NaAc); Quantitative check for insulin sensitivity (QUICKI).

The plasma insulin and 1-hour postload glucose levels but not fasting blood glucose significantly increased (p<0.05) in animals with PCOS compared to the control animals, which were significantly reduced (p<0.05) in PCOS+NaAc animals compared to the untreated PCOS animals. In addition, insulin sensitivity as determined by QUICKI significantly (p<0.05) decreased in animals with PCOS compared to the control animals and administration of sodium acetate significantly increased (p<0.05) insulin sensitivity in PCOS+NaAc animals compared to the untreated PCOS animals (Fig 3).

### 3.5 Impact of sodium acetate on triglyceride, total cholesterol and free fatty acid in LET-induced PCOS rat model

**Fig 4:**
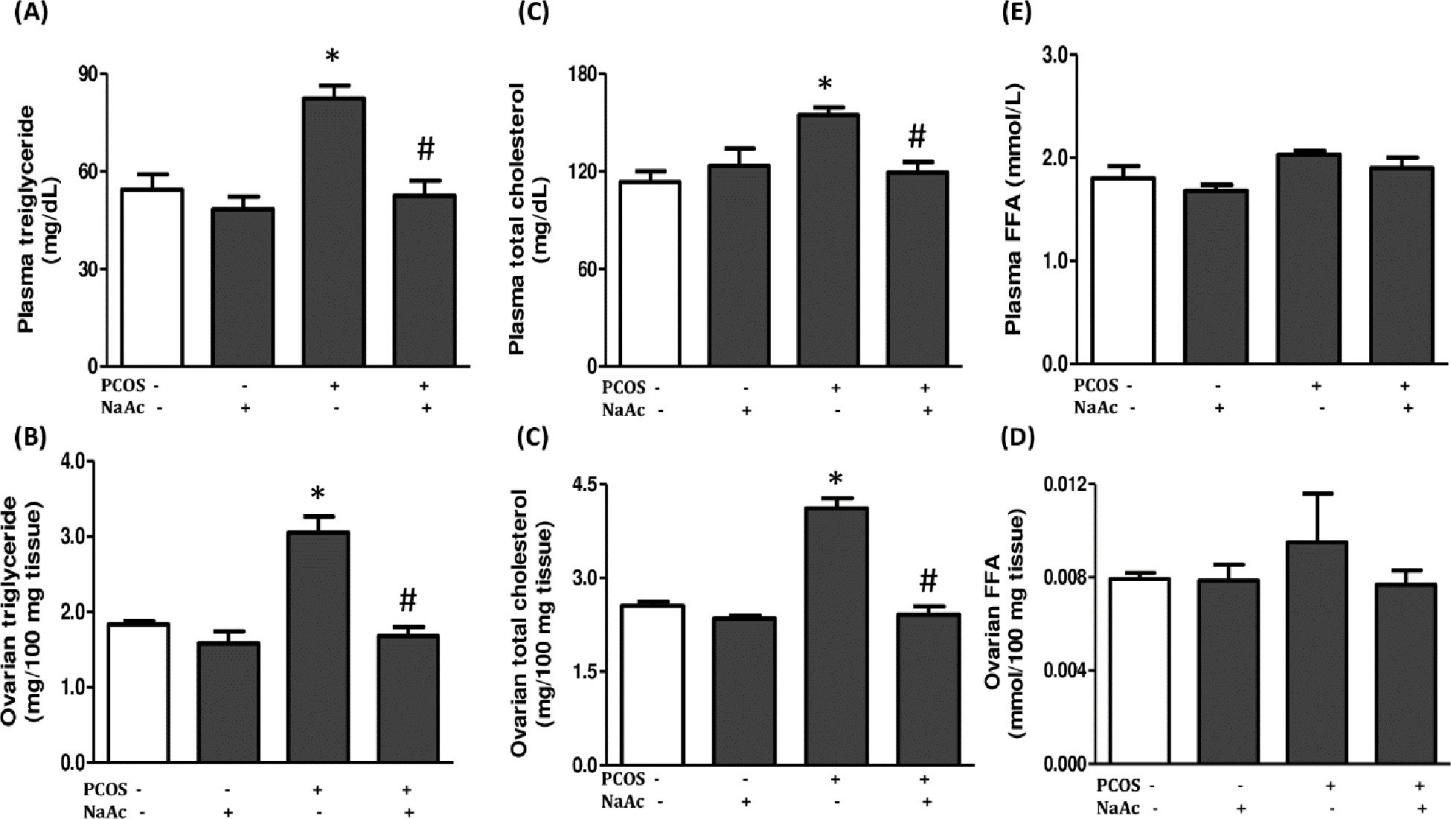
Impact of sodium acetate on plasma and ovarian triglyceride (a, b), total cholesterol (c, d) and free fatty acid (e, f) in LET-induced PCOS rat model. Data are expressed as mean ± SD. n = 6. Data were analyzed by one-way ANOVA followed by Bonferroni *post hoc test*. (*p<0.05 VS. CTL; ^#^*p*<0.05 VS. PCOS). Control (CTL); Polycystic ovarian syndrome (PCOS); Sodium acetate (NaAc); Free fatty acid (FFF).

The plasma and ovarian triglyceride and total cholesterol but not free fatty acid increased significantly (p<0.05) in animals with PCOS compared to the control animals, which were significantly decreased (p<0.05) in PCOS+NaAc animals compared to the untreated PCOS animals (Fig 4).

### 3.6 Impact of sodium acetate on lipid peroxidation, nuclear factor erythroid-derived 2–like 2 and antioxidant capacity in LET-induced PCOS rat model

**Fig 5:**
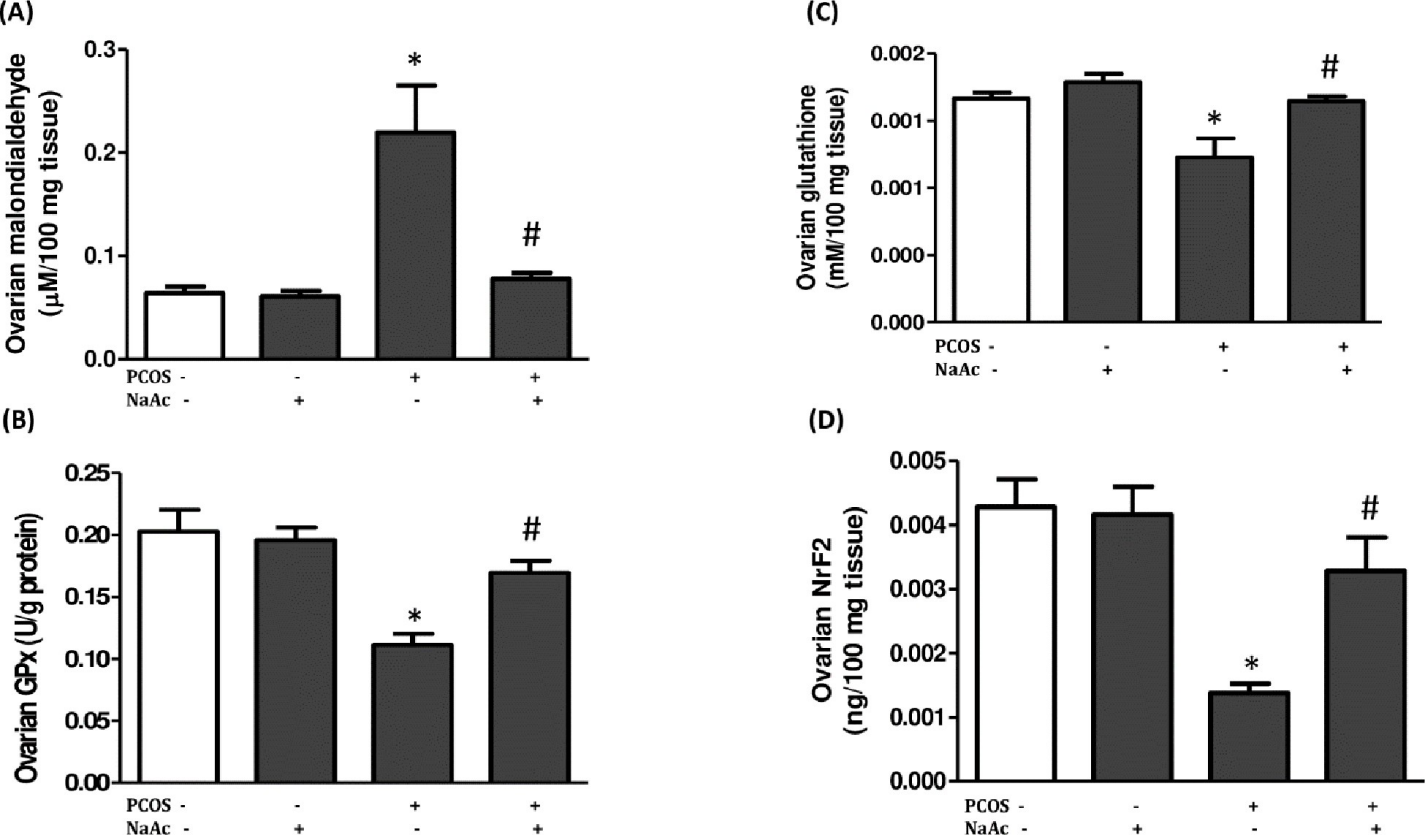
Impact of sodium acetate on ovarian malondialdehyde (a), glutathione peroxidase (b), reduced glutathione (c) and Nrf2 (d) in LET-induced PCOS rat model. Data are expressed as mean ± SD. n = 6. Data were analyzed by one-way ANOVA followed by Bonferroni *post hoc test*. (*p<0.05 VS. CTL; ^#^*p*<0.05 VS. PCOS). Control (CTL); Polycystic ovarian syndrome (PCOS); Sodium acetate (NaAc); nuclear factor erythroid-derived 2–like 2 (Nrf2).

The ovarian MDA increased while Nrf2, GPx and GSH decreased (p<0.05) in animals with PCOS compared to the control animals, which were significantly decreased (MDA) and increased (Nrf2, GPx and GSH) (p<0.05) respectively in PCOS+NaAc animals compared to the untreated PCOS animals (Fig 5).

### 3.7 Impact of sodium acetate on tumor necrosis factor-α and histone deacetylase in LET-induced PCOS rat model

**Fig 6:**
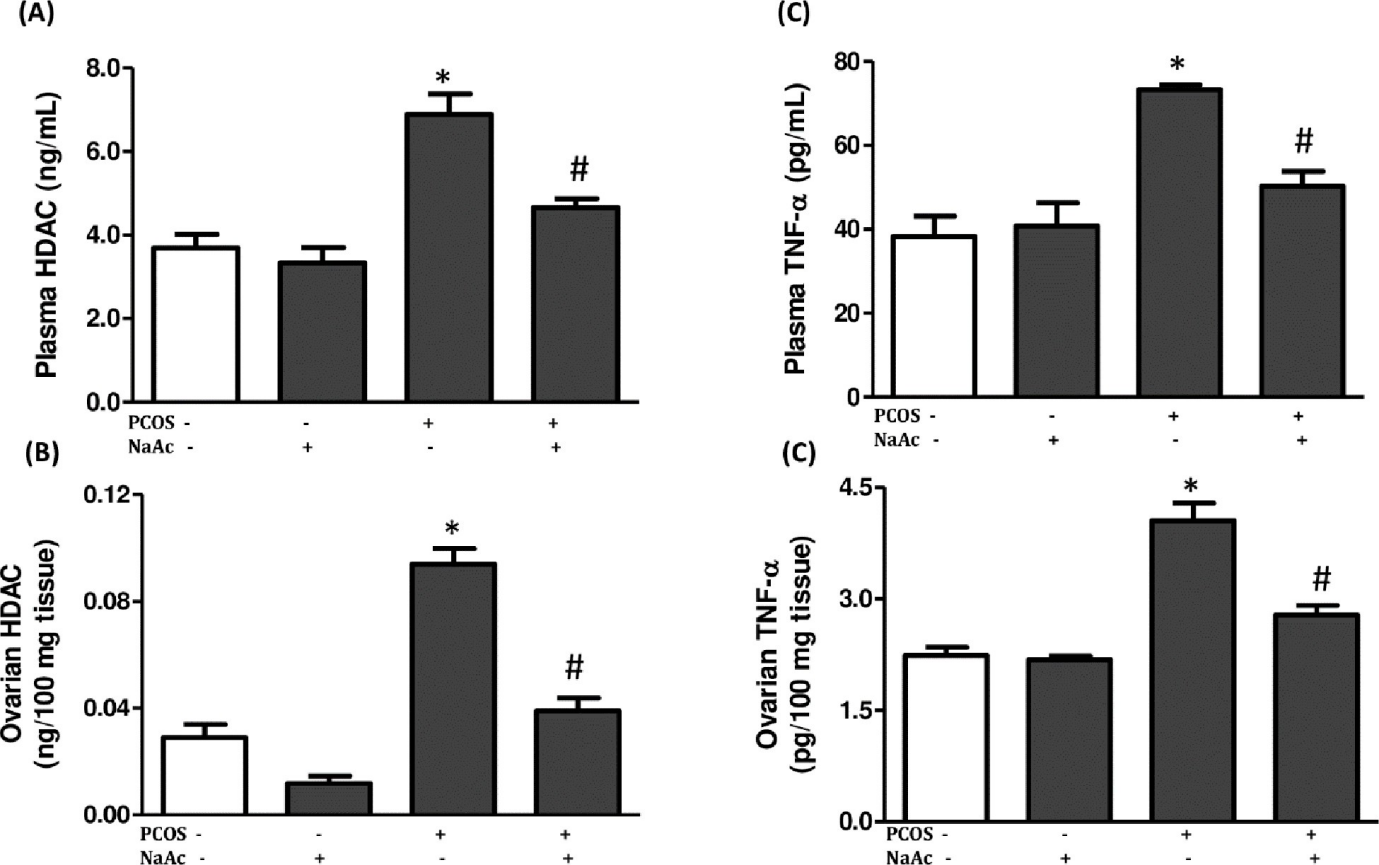
Impact of sodium acetate on plasma and ovarian histone deacetylase (a, b) and tumor necrosis factor-α (c, d) in LET-induced PCOS rat model. Data are expressed as mean ± SD. n = 6. Data were analyzed by one-way ANOVA followed by Bonferroni *post hoc test*. (*p<0.05 VS. CTL; ^#^*p*<0.05 VS. PCOS). Control (CTL); Polycystic ovarian syndrome (PCOS); Sodium acetate (NaAc); Histone deacetylase (HDAC); Tumor necrosis factor-α (TNF- α).

The plasma and ovarian levels of TNF-α and HDAC increased significantly (p<0.05) in animals with PCOS compared to the control animals. However, administration of sodium acetate significantly reduced the levels of TNF-α and HDAC in PCOS+NaAc animals compared to the untreated PCOS animals (Fig 6).

## 4. Discussion

The main result from the present study demonstrates that HDACi, acetate attenuates ovarian dysfunction in experimentally induced PCOS rat model. The study in addition showed increased body and organ weights, 1-hour postload glucose, reduced insulin sensitivity and caused hyperinsulinemia. Besides, PCOS led to significant elevations in plasma and ovarian TG, TC but not FFA levels, lipid peroxidation, heightened TNF-α, diminished GSH/GPx contents and perturbed ovarian histomorphology that are associated with modulations in HDAC/Nrf2 levels. The dysmetabolic characteristics of PCOS model in this study are consistent with hormonal disruptions including increased plasma testosterone, LH/FSH ratio, and depleted 17-β estradiol and SHBG levels. Nevertheless, acetate attenuated IR and ovarian lipotoxic/pro-inflammatory events and restored reproductive hormones balance in LET-induced PCOS rat model.

Polycystic ovarian syndrome is a serious female reproductive aberration that is worsened by attendant metabolic derangements with higher predisposition to CVD and ovarian cancers. Alterations in ovarian endocrine function, particularly androgen production serves a prominent role in the pathogenesis of infertility and metabolic syndrome in PCOS women [3]. Data from this study showed a significantly elevated plasma testosterone level and LH/FSH ratio with a decrease in 17-β estradiol level in letrozole-induced PCOS model compared with non-PCOS control. Studies show that hyperandrogenism in PCOS women is in part owing to neuroendocrine (hypothalamic-pituitary-ovarian-axis) dysregulation [32], ovarian cytochrome p450 aromatase inhibition [33] and/or an alternative steroid backdoor pathway [34]. The neuroendocrine basis of testosterone excess in PCOS is attributable to increased LH pulse which is confirmed in the present study by an elevated LH relative to FSH level in consonance with previous studies [35]. Moreover, that plasma estrogen level was decreased in this model can be related to failure of aromatization of testosterone by aromatase enzyme [36], although estimation of aromatase activity was not in the scope of this experiment. Data also indicate a reduction in plasma steroid hormone binding globulin (SHBG) level compared with control. Literature supports that low SHBG increases the amount of unbound/free testosterone, and thus promotes its bioavailability and action in PCOS patients [10].

Although not all PCOS women are overweight or obese, excessive weight gain with PCOS worsens the metabolic profile of this subclass of women [6]. Body weight gain was significantly increased in experimental PCOS in the present study with similar observation being previously reported [28]. Our data further showed that there was impaired oral glucose tolerance highlighted by elevated one-hour postload glucose in rats induced with PCOS. One-hour postload glucose is shown to be a reliable independent index of IR and represents a snapshot of delayed/altered glucose uptake by insulin-sensitive cells [37]. Moreover, insulin resistance was validated in the present study using QUICKI, which revealed significant reduction in insulin sensitivity. There was also a consequential increase in fasting plasma insulin level without a significant increase in fasting glycemia compared with control. Though a compensatory effort by the beta-cells in order to increase peripheral glucose disposal, elevated insulinemia contributes to the battery of PCOS disturbances via induction of errant androgen biosynthesis/availability and visceral fat lipolysis [7].

Studies have not sufficiently clarified the causal relationship between IR and ovarian dysfunction in PCOS. However, it is well known that both hyperinsulinemia and hyperandrogenism favor lipid dysregulation and abnormal distribution of fats that could lead to non-adipose tissue lipid accumulation. We provide some evidence in this study that PCOS elicited peripheral deposition of lipids with significant increase in plasma and ovarian TG and TC contents. Plasma and ovarian FFA levels were however not significantly increased compared to control. The adverse circulating and intra-ovarian lipid status correlates with increased ovarian weight in the current model. Wang et al. reported similar observation on increased ovarian weight in dehydroepiandrosterone-induced rat PCOS model but did not give information on intra-ovarian lipid status [38]. Excessive lipid deposition in the ovary could impair cellular fat oxidation as energy influx would exceed expenditure, causing accumulation of highly reactive lipid metabolites resulting in lipotoxicity [39]. Part of this metabolic oddity is the reaction of lipids with free radicals and superoxides originating from mitochondrial oxidative processes to produce MDA which can cause further damage to ovarian cells [40]. In line, ovarian MDA level was significantly elevated in this PCOS model compared to controls.

Furthermore, our data revealed increased level of TNF-α in the plasma and ovaries of experimental PCOS rats. The pro-IR/inflammatory cytokine is secreted by activated immune cells recruited into lipotoxic plasma and ovarian milieu. Elevated TNF-α level was reported in the follicular fluid of obese-PCOS women [11] and was suggested to be the link between PCOS with ovarian cancers [12]. Also, in the present study, there was a significant diminishing of ovarian GSH and GPx antioxidants which could have emanated from excessive ROS production. Usually, oxidative stress is caused by an imbalance between highly reactive peroxides/free radicals and antioxidants’ availability or capacity to nullify them. Cells adapts to oxidative stress by increasing the expression of Nrf2 in order to promote transcription of antioxidant genes. The severe oxidative/inflammatory stress during lipotoxicity impairs antioxidant function by decreasing Nrf2 level [16]. The observed decrease in ovarian GSH and GPx contents in this study is further corroborated by a significant reduction in Nrf2 level in PCOS rats. This finding is in line with that of recent studies [41,42].

Furthermore, evaluation of histomorphological indices was conducted to verify whether the endocrine-metabolic deviations observed in our PCOS model alters ovarian histoarchitecture. There were degenerated ovarian follicles and disrupted antrum, thecal cells, granulosa cells and oocyte in PCOS rats compared with controls. Besides, the number of degenerated follicles was significantly higher in PCOS animals compared with control. Existing studies in agreement with ours documented varying degrees of histomorphological distortions in the ovary of letrozole-induced PCOS models [8,43]. Changes at certain epigenomic marks such as deacetylation alter the transcriptional activity of key proteins involved in the regulation of ovarian metabolic function [13]. In the present study, we investigated whether HDAC mediates the dysmetabolic/lipotoxic/inflammatory and histopathological features in the PCOS model. There was a significant increase in plasma and ovarian HDAC levels in experimental PCOS rats compared with control. A previous study gave evidence that HDAC down-regulated Nrf2 transcriptional activity permitting oxidative stress and inflammation in diabetic mice [15]. Others found that pharmacological HDAC inhibition switches on the Nrf2 pathway [41,42]. Similarly in previous investigations, we observed that HDAC level correlates with pro-inflammatory TNF-α level in diabetic Wistar rats [24,25]. However, our present finding is the first to demonstrate the involvement of HDAC in TNF-α/Nrf2 modulation-mediated ovarian dysfunction in experimentally induced PCOS.

Improvement of body weight and insulin sensitivity is an important therapeutic goal in PCOS management that dietary restriction and exercise may not be sufficient to achieve. Hence the search for best effective treatment option is ongoing. Metformin is the first line drug to improve insulin sensitivity and decrease insulin contribution to hyperandrogenism in PCOS patients [19]. However, there has been some variation in terms of response to metformin treatment amongst PCOS women, hence, a more efficient and inclusive therapy is urgently needed [4]. Interestingly, acetate treatment of PCOS rat significantly suppressed plasma and ovarian HDAC with attendant abrogation of pro-IR/inflammatory TNF-α effect, and restored Nrf2 and related antioxidant defense (GSH and GPx). Administration of acetate in the present study also decreased hyperlipidemia, ovarian lipid accumulation, lipid peroxidation, hyperinsulinemia, glucose intolerance, insulin insensitivity, body weight gain, ovarian weight, histomorphological disruptions, plasma testosterone and LH/FSH ratio while 17-β estradiol and SHBG levels were increased in experimentally induced PCOS. HDAC inhibition-dependent resolution of ovarian inflammatory/oxidative stress and altered histology by acetate in this study confirms the findings recently reported by our laboratory in diabetic rats [25]. Meanwhile, this study suggests that acetate through HDAC suppression protects against ovarian dysfunction in LET-induced PCOS rat model. However, the present study is not without some limitations, in such that functional evaluations such as rate of implantation and fertility were not investigated. Nevertheless, this study forms a relevant justification for future molecular investigation and perhaps, provides a promising clinical relevance for the management of PCOS.

## 5. Conclusion

Altogether, the present results suggest that acetate protects ovarian function with evidence of normal growing follicles and enhanced circulating 17-β estradiol by inhibition of HDAC. Therefore, supplementation with acetate is a promising non-pharmacological protective/therapeutic agent for PCOS individuals.

## Supporting information

S1 Data (PDF)
