# Supplementary material for: Short chain fatty acid, acetate restores ovarian function in experimentally induced PCOS rat model
Source: PLoS One. 2022 Jul 26;17(7):e0272124. doi: 10.1371/journal.pone.0272124 (PMC9321379; doi:10.1371/journal.pone.0272124)
Supplement: S1 Data — (PDF) [file pone.0272124.s001.pdf]

Fig 1

	C	N	P	K		C
1	19	23	39	18	1	1.764438
2	24	20	37	22.4	2	1.604034
3	20	19	38.6	20	3	1.924852
4	16.5	24	41	22.3	4	1.668196
5	19.7	12	38.5	22.3	5	1.86068
6	19.8	17	37	29	6	1.764427

Fig 3

	C	N	P	K		C
1	70.95	73.8	82.8	97.2	1	86.4
2	88.2	69.4	78.4	73.8	2	79.2
3	82.8	70.2	79.2	66.95	3	82.8
4	82.8	73.8	84.6	64.8	4	81
5	80.55	71.55	81	75.15	5	75.6
6	78	70.55	80	73	6	81

Fig 4

	C	N	P	K		C
1	67.01031	52.57732	97.73196	68.04124	1	1.74375
2	60.79038	38.14433	87.73196	59.10653	2	1.988125
3	63.19586	47.76632	83.23024	56.35739	3	1.834625
4	52.23368	56.70103	79.10653	51.54639	4	1.85
5	37.45705	36.42612	75.94501	43.64261	5	1.745625
6	45.01718	58.41924	70.79037	35.73883	6	1.845625

Fig 5

	C	N	P	K		C
1	0.064031	0.060847	0.219643	0.077908	1	0.19412
2	0.054235	0.044031	0.239439	0.078724	2	0.202562

3	0.087908	0.057806	0.384847	0.074643	3	0.2532
4	0.057806	0.063418	0.195051	0.09301	4	0.19412
5	0.06648	0.077602	0.154745	0.084847	5	0.20257
6	0.053724	0.061378	0.124133	0.058316	6	0.1688

Fig. 6

	C	N	P	K		C
1	3.73	3.091	8.015	3.939	1	0.02836
2	3.464	2.194	7.194	4.656	2	0.02315
3	3.687	3.337	6.891	4.6596	3	0.028857
4	4.601	3.96	5.367	4.964	4	0.04291
5	2.601	3.189	7.639	5.177	5	0.028858
6	4.038	4.253	6.237	4.562	6	0.02101

N	P	K
1.295206	3.31718	1.771217
1.64876	3.383259	1.937269
2.296048	3.964757	1.9557
1.648445	3.177533	1.955738
1.354079	2.643171	1.955719
1.648	3.41718	2.158671

N	P	K
81.56	99.19999	79.2
82.8	117.6	90
80.19999	105.22	77.4
82.8	113.2	75.6
77.4	96.4	66.6
84.6	99.7	77.76

	C	N
1	1.68	1.59
2	1.44	1.533
3	1.57	1.61
4	1.578	1.49
5	1.57	1.769
6	1.58	1.611

N	P	K
1.136875	2.945625	1.95625
1.275	2.78625	1.245625
1.58175	3.05175	1.677052
1.70125	2.98625	1.619034
1.839375	3.860625	1.870159
1.95625	2.68	1.694191

	C	N
1	120.1923	99.03846
2	131.7308	102.8846
3	124.0385	131.7308
4	114.4231	129.6154
5	102.8846	108.6538
6	86.53846	168.2692

N	P	K
0.17724	0.13632	0.17724
0.195808	0.11104	0.169536

	C	N
1	0.001779	0.00173
2	0.001561	0.001827

0.17724	0.10256	0.20128
0.22788	0.111	0.14348
0.18568	0.1112	0.15908
0.211	0.09412	0.1666

3	0.001665	0.001789
4	0.001597	0.001742
5	0.001767	0.001997
6	0.001621	0.001646

N	P	K
0.00589	0.093842	0.048773
0.011696	0.085013	0.038841
0.01927	0.08376	0.03376
0.00944	0.107823	0.027823
0.011698	0.093843	0.038843
0.01219	0.098773	0.045013

	C	N
1	35.14803	28.81548
2	52.61956	31.80711
3	31.38949	35.98326
4	33.82686	50.53149
5	38.2459	56.79572
6	38.24599	40.78661

P	K
3.25	1.503
4.854	1.79
3.74	1.67
3.94	1.69
2.933	1.73
3.743	1.67

	C	N	P	K
1	1.180012	1.280113	0.932551	1.239688
2	1.242093	1.287653	0.88294	1.099683
3	1.211489	1.24634	0.844737	1.207185
4	1.21	1.272313	0.83947	1.266133
5	1.246872	1.25	0.810899	1.275203
6	1.178469	1.141622	0.757824	1.155219

P	K
149.0385	125.9615
150.9615	127.1154
139.4231	119.2
162.5	123.6538
152.8846	119.18
172.1154	100

	C	N	P	K
1	2.76622	2.46622	4.547826	2.26614
2	2.553598	2.351598	4.115678	2.413175
3	2.44444	2.31444	3.878524	2.487484
4	2.43493	2.23493	3.867984	2.86693
5	2.471805	2.291805	4.458234	2.097497
6	2.650593	2.450593	3.825824	2.347826

P	K
0.000877	0.001536
0.001267	0.001694

	C	N	P
1	0.00479	0.00522	0.00168
2	0.00427	0.00334	0.001374

0.001227	0.001646
0.00173	0.00173
0.001064	0.001588
0.001197	0.001682

3	0.00336	0.004162	0.00112
4	0.00522	0.00452	0.00112
5	0.00428	0.004163	0.001375
6	0.00375	0.00357	0.00158

P	K
71.34394	52.96886
72.66512	36.33256
72.66512	55.96049
76.33256	51.78433
73.25168	54.15942
73.25169	50.24113

	C	N	P
1	2.23715	2.180934	4.057064
2	2.10625	2.36414	4.01701
3	2.34634	2.12671	3.94849
4	2.53304	2.09901	3.742
5	2.31402	2.18923	4.95269
6	1.8861	2.12558	3.62513

	C	N	P	K	
1	1.642857	1.678571	2.089	1.785714	1
2	2.035714	1.5	2.04632	1.571429	2
3	2.25	1.642857	2.107143	1.892857	3
4	1.535714	1.785714	1.928571	2	4
5	1.535714	1.571429	1.928571	1.821429	5
6	1.8	1.892857	2.087	2.321429	6

K

0.003287

0.00434

0.00399
0.00264
0.003288
0.00218

K
2.781966
2.62001
2.78536
3.11772
2.99869
2.38805

C	N	P	K
0.007857	0.007857	0.0075	0.006786
0.007917	0.007857	0.0095	0.007678
0.0075	0.006786	0.006786	0.006429
0.0078	0.010357	0.009643	0.008571
0.008929	0.007857	0.0175	0.007678
0.0075	0.006429	0.006071	0.008929

	C	N
1	0.007857	0.007857
2	0.007917	0.007857
3	0.0075	0.006786
4	0.0078	0.010357
5	0.008929	0.007857
6	0.0075	0.006429

P	K
0.0075	0.006786
0.0095	0.007678
0.006786	0.006429
0.009643	0.008571
0.0175	0.007678
0.006071	0.008929
